# Cerebellar Abnormalities: A Component of Autism Pathophysiology

**DOI:** 10.3390/medicina62030435

**Published:** 2026-02-25

**Authors:** Rekha Jagadapillai, Idil Tuncali, Naveen Nagarajan, Gregory Barnes, Evelyne Gozal

**Affiliations:** 1 Department of Pediatrics, Pediatric Research Institute (PRI), University of Louisville, Louisville, KY 40202, USA; 2 University of Louisville Autism Research Center, University of Louisville, Louisville, KY 40220, USA

**Keywords:** autism, cerebellum, astroglia, autism-linked genes, autism signaling pathways, neurodevelopmental disease

## Abstract

*Background and Objectives*: Autism spectrum disorder (ASD) is a prevalent and largely idiopathic developmental disorder with relatively widespread etiology. Currently, there are no validated diagnostic or screening biomarkers for ASD, besides addressing the associated comorbidities. ASD is primarily diagnosed based on behavioral, motor, and cognitive characteristics. Until recently, although the cerebellum was particularly implicated in motor control, it was under-researched for its potential role in the development of ASD. However, cerebellar circuitry is altered in ASD, impacting its brain interconnections, affecting brain development, as well as social and behavioral outcomes associated with ASD. *Methods*: We reviewed the potential role of the cerebellum in ASD, particularly how its dysfunction during development or its early postnatal injury may impact on the maturation of other connected circuits and play a role in the development of core ASD symptoms. *Results*: Based on the literature, we addressed cerebellar changes that may alter synaptic pruning, immune cells’ function, neurotransmitters, blood–brain barrier permeability, and potential signaling pathways involved in ASD, and how these changes may interplay to contribute to ASD pathophysiology. *Conclusions*: Further research is needed to understand these interactions that may provide novel therapeutic options specifically targeted at the cerebellum.

## 1. Introduction

Autism Spectrum Disorders (ASD) are neurodevelopmental diseases of varying severity, resulting from complex genetic, environmental, and immunological interactions. ASD is diagnosed by atypical social behavior and interactions, limited intellectual and interest skills, repetitive/stereotypic behaviors, and variable levels of cognitive and intellectual impairments [1]. Individuals with ASD may also present with other symptoms, such as seizures, anxiety, apraxia of speech, sleep disorders, and gastrointestinal complications. According to the CDC, about 1 in 44 children have been identified with ASD, corresponding to estimates from the CDC’s Autism and Developmental Disabilities Monitoring (ADDM) network [2]. While most cases are idiopathic, multiple genetic mutations were identified in patients and are widely studied in animal models, also implicating immune dysregulation and environmental factors, as all of these could interact to bring about ASD [1,3,4]. Identification of saliva biomarkers, using proteomics or RNA-based methodologies, offers a potential non-invasive early diagnosis tool. However, some protocol standardization is still required and, given the multifactorial etiology of ASD, specificity and sensitivity remain to be validated [5,6].

Therefore, there are currently no validated biomarkers for diagnosis or screening for ASD, besides addressing the related comorbidities. Therefore, understanding the fundamental mechanisms of the disease is of utmost importance to develop ASD-specific targeted treatment strategies.

Most studies have focused on various neural networks associated with social behavior, language, and cognition. Postmortem studies of patients with ASD, with genetic predisposition and showing clinical evidence, and of animal models such as Tsc1 mutant mice, have implicated cerebellar development and dysfunction in ASD etiology [7,8,9]. The cerebellum, previously mostly associated with motor functions, is now recognized for its involvement in cognitive functions, due to its complex connections with other brain areas. These functions, extensively studied in attention deficit disorders (ADD), include affective, emotional, and attentional control disorders; language and memory difficulties; impaired behavioral inhibition; environmental exploration; and social skills [10,11,12,13].

Cerebellar structural, functional, and neurochemical abnormalities have been identified in ASD, and lesions in particular cerebellar sites yield effects all throughout the brain via network connections [1,11,14]. Cerebellar dysfunction during development may affect the maturation of other connected cerebral circuits that have been shown to regulate several ASD-relevant behaviors [9,12,15]. Multiple animal models of early life cerebellar damage display ASD behavioral symptoms. Indeed, cerebellar defects, if they arise by birth or during early postnatal period, are often sufficient to induce ASD behavioral symptoms [7].

**Figure 1:**
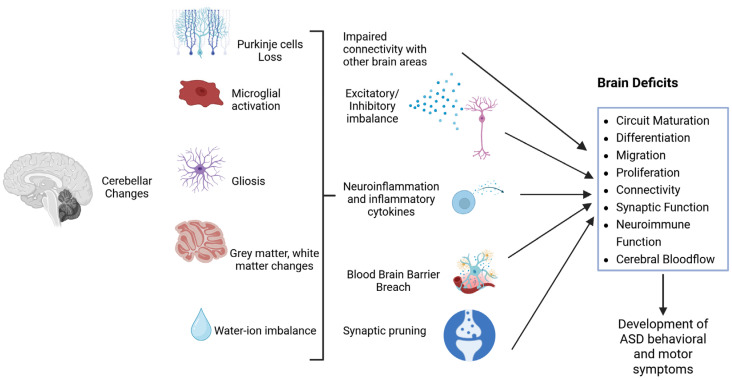
ASD-associated cerebellar alterations that may lead to ASD symptoms development. (Created with BioRender.com).

This review article offers a comprehensive overview of different aspects of cerebellar involvement with respect to brain development and function, enabling the onset of ASD (Figure 1), highlighting earlier studies and new potential areas of research. Thorough understanding of the cerebellar function in ASD may lead to novel strategies regulating the underlying mechanisms to elaborate treatments that are more precisely aimed at cerebellar dysfunction in individuals with ASD.

## 2. Cerebellum Structure and Function

The highly conserved brain structure and neuronal developmental trajectory of the vertebrate cerebellum develop following a conserved chronological sequence of neurogenesis, preserving its function and internal circuitry [16]. During evolution, the size of the mammalian cerebellum expanded when compared to the other areas of the brain, especially the cerebellar posterior lobe, which is important for the processing of cognitive, as well as language skills [1,17,18,19]. While the cerebellum occupies just 10% of the total brain volume, it contains more neurons than the rest of the brain because of its substantial number of granule cells [20,21].

The orderly spaced parallel grooves of the cerebellar cortex are organized in three layers: the outer molecular layer (ML), Purkinje cell layer (PCL) and the inner granular layer (GL). The neuronal organization of Purkinje cells (PC) and granule cells (GC) brings about an immense and complex signal processing potential. However, the output signal from the cerebellar cortex goes out through a set of cells, called cerebellar deep nuclei, sitting in the white matter of the cerebellum [22]. Mossy fibers, climbing fibers, and parallel fibers are the three types of axons that play major roles in the cerebellar circuit. The molecular layer, the outermost layer of the cerebellar cortex, is comprised of PC dendritic branches and parallel fibers, which are the axons of the granule cells. The molecular layer contains two types of cells, the stellate cells and the basket cells, which are inhibitory interneurons forming GABAergic synapses with PC dendrites [21]. PC are unique neurons in the brain, with large cell bodies arranged into a single cell layer of the cerebellar cortex. The PC dendrites receive inputs from parallel fibers, estimated around 200,000 dendritic spines on a single human PC, and are inhibitory in nature [21]. Loss of PC, consistent with impaired cerebellar function, is typically identified in ASD and may contribute to the excitatory/inhibitory imbalance that characterizes the disorder [15,23]. Thus, PC play a critical role in providing downstream cerebellar nuclei with efferent inhibitory output [24,25]. Granular cells are small, seen in large number in the human brain, estimated around 50 billion in total number, and are mainly excitatory in nature, as glutamate is their neurotransmitter [21]. Granular cells receive input from mossy fibers, while PCs receive input from climbing fibers.

Cerebellar deep nuclei consist of three nuclei embedded within the white matter, the fastigial (medial) nucleus, the interposed nucleus, and the dentate (lateral) nucleus. These nuclei together form the exclusive output of the cerebellum, and it has been estimated that the total number of cerebellar deep nuclei neurons in the cerebellum is about 50–100,000 [26], including both excitatory projection neurons [27,28,29] and inhibitory projection neurons [30,31]. Therefore, disruption of GC/PC and cerebellar deep nuclei output may contribute to excitatory/inhibitory imbalance underlying ASD neuropathophysiology.

The cerebellar deep nuclei are closely associated with the cerebellar functions related to the sensorimotor region, limbic system, etc. In addition to their role in motor function, it has been reported that the cerebellar deep nuclei are also involved in cognitive and linguistic functions [32], as evidenced by neuropsychological, neurophysiological, and imaging studies.

Evidence shows that the cerebellum may coordinate communication and contribute to sensorimotor deficits in ASD [9,15,25,33]. For instance, while the primary cause of apraxia is damage to the cortex, cerebellar damage can also contribute to apraxia of speech [32,34]. A recent study from Milton S. Hershey Medical Center recognizes apraxia as a common incidence in ASD, involving both speech and communication. Apraxia of speech affects children’s brain pathways accountable for performing movement associated with speech production and their ability to align motor movements to deliver speech, despite having fully functional muscles [1]. In a previously reported study, 64% of children with ASD diagnosis also exhibited apraxia of speech, and 37% of children with an apraxia diagnosis also had ASD [35]. Because of the failure in coordinating tongue, lips, mouth, and jaw, the same word would be pronounced differently every time it was spoken [35].

Localized lesions in the cerebellum yield effects throughout the brain via its network connections (Table 1) [1]. Patients with cerebellar damage failed to control their thought processes, as the cerebellum plays a crucial role in regulating cognitive processes [10,36]. Although ASD is behaviorally defined, brain structural differences may be implicated. In neuroimaging and neuromodulation studies, specific sub-cerebellar region differences have been associated with ASD, both in human and animal studies [9,12,23,25]. Van Overwalle et al. reported decreased cerebellar volume in the posterior vermis, bilateral Crus II, and right VI and Crus I/II in children with autism, correlating with social interaction and communication scores. Reduced gray matter volume was found in the posterior vermis, Crus I/II, inferior cerebellar vermis (lobule IX), left lobule VIIIB, and right Crus I in autism. Children with ASD also displayed differences in cerebellar activation in lobule VII, including Crus I/II. Among other differences, cerebellar volume and reduced gray matter volume, white matter, and activation in specific cerebellar regions have been observed in individuals with ASD compared to controls [9,12,37,38,39].

Studies also documented a significant reduction in Purkinje cells in the brains of individuals with autism [40,41,42,43]. Understanding how these cerebellar dysfunctions are associated with neuroinflammation and neuroimmune alterations seen in ASD would contribute to targeted intervention strategies.

## 3. Cerebellar Astroglial Involvement in ASD

Reports from our laboratory and others, including postmortem and genomic studies, demonstrated neuroinflammation with increased pro-inflammatory cytokines, as well as astrocytic and microglial activation, in brains of animal ASD models, such as the interneuron-specific Sema3F KO mouse model, and in patients with ASD [44,45,46,47].

### 3.1. Cerebellar Astrocyte Involvement

Astrocytes, the most numerous glial cell type, accounting for one third of brain mass, are involved in the maintenance of the blood–brain barrier (BBB), regulation of water, ion homeostasis, and amino acid neurotransmitter metabolism, as well as energy and nutrient support of neurons. Neuron–glia bidirectional communication is associated with the proliferation, migration, and differentiation of neural precursor cells and is essential for normal functioning of the brain during early neurodevelopment and throughout life. Altered expression of astroglial markers, such as GFAP, aquaporin-4 (AQP4), and connexin 43 (CX43), has been reported in postmortem studies of patients with ASD [48,49,50]. During neuroinflammation, GFAP expression is upregulated when astrocytes are hypertrophic and proliferate. Other studies in postmortem ASD brains have also reported an upregulation of *GFAP* gene expression, as well as an increase in GFAP protein level in the cerebellum [46,48,49,51,52]. Mounting evidence has illustrated that glial cells play a key role in synaptic pruning through phagocytosis in health and disease [53,54,55,56,57]. To uncover the involvement of glial phagocytosis in synaptic pruning, Morizawa et al. created a genetic strategy for visualizing phagocytic events [58]. In naive healthy mouse cerebellar cortex, they found that Bergmann glia (BG) have a high phagocytic capacity, and the BG engulfment of neuronal structures, spines, and dendrites was characterized by three-dimensional electron microscopy (3D-EM) analysis. Tissue examination of mice undergoing cerebellum-dependent motor learning revealed enhancement of nibbling of both presynaptic and postsynaptic structures by BG, including postsynaptic spine volume reduction [58].

CX43 is a major protein component of the astrocytic gap junctions and is primarily expressed in BG in the cerebellum. CX43 is responsible for regulating cellular growth and cell–cell adhesion, which also allow for gap junction intercellular communication between cells to regulate cell death, proliferation, and differentiation. Studies reported a higher expression level of CX43 in patients with ASD [59], which could suggest enhanced glial–neuronal communication in the brain. CX43 can be involved in influencing astrocyte neurotransmitter transport, thus inhibiting hyperactivation of the nervous system [60]. CX43-mediated gap junction overactivation can trigger excitotoxicity, neuroinflammation, and other pathological conditions [61,62]. Excitation/Inhibition (E/I) imbalance has been observed in patients with ASD, in whom there is generally less inhibition to balance overexcitation [63,64]. Abnormal CX43 expression causes (E/I) imbalance and excitotoxicity, which, in turn, can substantially hamper synaptic pruning through triggering synapse loss, disturbing neural connections, causing network dysfunction, and contributing to neurological disorders [65,66]. However, conflicting findings report that CX43 decreased as a result of AQP4 depletion [67]. Earlier findings enable us to assume that correcting the AQP4 changes in the brain could also normalize function and expression of CX43 in the gap junctions.

We hypothesize that reactive astrocytes and alterations in GFAP, AQP4, and CX43 expression in the cerebellum not only compromise the neuron–glia communication but also facilitate and aggravate the effects of neuro-inflammation and edematous condition, as well as hinder synaptic sprouting during early development. Cerebellar abnormalities may also be responsible for dysfunctions within the motor system that are associated with autism. It is therefore important to understand the effects of astrocytes, and especially how the alterations in any of the GFAP, AQP4, or CX43 markers in the cerebellum may contribute to the development of therapeutic advances in patients with ASD.

AQP4 is widely expressed in astrocyte terminal feet enwrapping blood vessels that constitute the blood brain barrier (BBB) and is the most abundant water channel in the CNS. AQP4 has many functions, especially in a developing brain, such as accelerating cell migration by facilitating the transmembrane water fluxes that mediate migrating cells’ rapid changes in shape and volume as they move through a developing brain [68]. Decreased cerebellar AQP4 expression may mean that the cell structure, cell volume, and ionic homeostasis are compromised.

In addition, AQP4 regulates the water transport in various organs, such as the formation of cerebrospinal fluid in the brain, of urine, and of aqueous humor of the eye [69,70,71]. As reported, water accumulation was greater in AQP4 knockout mice than in wild type mice in brain tumor edema and vasogenic edema [72,73]. Bloch et al. demonstrated that brain swelling developed at a faster rate in a mouse model of AQP4-null obstructive hydrocephalus [74]. Studies also report AQP4’s involvement in seizures, as seizure duration and intensity increased in AQP4-deficient mice [75]. This phenomenon is supported by studies showing that delayed K^+^ reuptake by AQP4-deficient astrocytes leads to neuroexcitation [75,76]. In addition to synaptic alterations, dysregulation of AQP4 leads to compromised brain homeostasis, BBB development, and BBB breach, which have been associated with autism and Fragile X through overlapping pathways of neuroinflammation and synaptic dysfunction [77].

Furthermore, several studies support the hypothesis that AQP4 plays a key role in regulating synaptic plasticity, including compelling data, suggesting a promising link between defective long-term potentiation (LTP) and the downregulation of glutamate transporter-1 (GLT-1) [78,79,80,81,82,83,84,85]. Zeng et al. and others have evidenced decreased GLT-1 expression in AQP4-null mice, leading to reduced glutamate uptake by astrocytes [85,86].

Another potential mechanism that could be linked to synaptic plasticity, as well as learning and memory impairment, is the decrease in BDNF signaling in AQP4-deficient mice [87,88]. Studies found that the release of mature BDNF leads to LTP [89] and the inhibition of LTD [90]; hence, the delayed LTP in AQP4^−/−^ mice was a remarkable finding [78]. These data suggest a tight control of BDNF release for either LTP or LTD and that BDNF release in adaptable synaptic plasticity may be modified by AQP4.

Broad opportunities remain for the development of AQP4-based diagnostics and therapeutics. Several anti-epileptic drugs, including zonisamide, lamotrigine, phenytoin, and topiramate, were also found to inhibit AQP4 [91]. In addition, 2-(nicotinamide)-1,3,4-thiadiazole (TGN-020) appears to be a more selective inhibitor and has demonstrated promising results in preclinical studies [92]. Inhibitors of AQP4 and small molecule aquaporin modulators are predicted to reduce brain swelling in cytotoxic edema, potentially offering neuroprotection following brain injury, ischemic stroke, epilepsy, infection, and neuroinflammation, and thus could also alleviate ASD symptoms.

### 3.2. Cerebellar Microglia, Macrophage, Monocyte, and Neutrophil Involvement

Previous reports have documented increased microglia and Bergmann glia reactivity within the PC layer and accompanying white matter in the brains of patients with ASD. Activated microglia are detected in the vicinity of degenerating PCs and granule cells [46]. The microglia and astrocyte activation extends beyond the cerebellum to mid-frontal and cingulate gyrus, as measured by the increased expression of cell surface major histocompatibility complex molecule HLA-DR and glial fibrillary acidic protein. Activated microglia and astrocytes are also accompanied by monocyte and macrophage accumulation. Such accumulation may underlie the phagocytic capacity of synaptosomes by human macrophages derived from peripheral blood monocytes and macrophage polarity [93]. Higher microglia activation in white matter is detected in individuals with ASD and a history of epileptic seizures, compared to the population with ASD but without epilepsy. The microglial activation results in increased production of several cytokines and chemokines, such as interleukin (IL-6), transforming growth factor beta 1 (TGFβ1), C–C motif ligand 2 (CCL2), and CCL17 [46,94,95,96]. Gene expression analysis of patients with ASD further confirms significantly increased and/or activated MET, NF-κB, IL-1 receptor, TOLL, and TNF receptor 2 immune system-related genetic pathways. Whether the altered immune activity is causal, or in response to the neuronal damage induced during early onset of ASD, is unknown [97]. Beyond the cerebellum, immune activation is also a hallmark factor that is compounded with elevated plasma cytokine levels (IL-1β, IL-6, IL-12, and TNFα), immunoglobulin levels, and complement proteins and chemokines (CCL2, CCL5, CCL11) within the periphery. The natural killer cells, monocytes, and T cells that show higher activation in response to immunological challenges disrupt cellular functions in patients with ASD in parallel to decreased production of regulatory cytokines such as TGFβ1 and IL-10, implying a shift in inflammatory profile [98,99,100,101]. The high production of cytokines also correlates with higher activation of monocytes, macrophages, mast cells, and microglia [102]. Applying CIBERSORT algorithm, Li et al. [103] showed that the ASD patient group exhibits a significantly higher estimated proportion of resting/activated dendritic cells, M0/M2 macrophages, and monocytes in children, and M0 macrophages, resting mast cells, and resting/activated NK cell group in adults, with a higher percentage or increasing trend of apoptosis in monocytes [104]. Preclinical animal models support clinical studies, especially with respect to monocyte infiltration [105] and neutrophil density [106]. We have previously shown increased microglial activation in the Sema3F-KO mouse model of autism [47]. The idiopathic BTBR autism mouse model that exhibits ASD phenotypes displays increased IL-33, IL-18, and IL-6 cytokines [107]. More recent results show that the cerebellum granular layer-specific knockout of PTEN that results in macrocephaly, motor coordination defects, cellular hypertrophy, and ASD phenotype in mice also shows enhanced microglia phagocytic capacity and aberrant microglia activation [108]. Beyond glial activation, children with ASD exhibit a higher neutrophil-to-lymphocyte ratio (NLR), primarily mediated by acute immune infections, autoimmune diseases [109], and superoxide dismutase-mediated dysregulated enzymatic antioxidant network [110]. In summary, both humans and mice with ASD phenotype show increased generalized and cerebellar microglia activation that is associated with altered cytokine and chemokine levels, enhanced monocyte and macrophage accumulation, and higher neutrophil density.

Several immune-regulating compounds have demonstrated a potential effect decreasing some of the inflammatory ASD symptoms. Several anti-inflammatory compounds, such as a COX2 inhibitor (Celecoxib) and inflammatory cytokines inhibitor (Pentoxifyllin), have been shown to improve social deficits, but have not yet been proven as significant therapies [111,112,113,114].

## 4. Neurotransmitter and Cerebellar Connectivity Associations with ASD

The neurobiological characteristics of ASD comprise abnormal synaptic connectivity, imbalance in excitatory inhibitory signaling, and alterations in neurotransmitters such as glutamate, gamma aminobutyric acid (GABA), dopamine, and serotonin [115,116]. Thus far, accruing evidence points to the hypothesis that fundamental features of ASD emerge from irregularities in the excitatory/inhibitory balance within neural circuits [117,118,119]. Among the neurotransmitters involved, the abnormal signaling of glutamate and GABA have been steadily reported to be the most affected in ASD (Table 2) [120,121,122]. A study by Purcell et al. reported a decrease in glutamatergic neurotransmission in the cerebellum of patients with ASD when compared to those of individuals considered neurotypical [51]. Both motor and cognitive impairments in ASD direct attention to an excitatory/inhibitory imbalance within the cerebellum [123] (Table 2).

### Cerebellar Coordination of Cerebral Activity Involvement in ASD

Among the three main layers of the cerebellar cortex, the outer molecular layer is composed of inhibitory neurons such as stellate cells and basket cells, and the Purkinje layer consists of inhibitory Purkinje cells. The inner granular layer is composed of both excitatory granule cells and inhibitory Golgi cells. The mossy fibers and the climbing fibers are the primary input pathways entering the cerebellum, where in the granular layer mossy fibers synapse on the dendrites of granular cells, whose axons lead to the molecular layer, where they form parallel fibers [124,125]. Mossy fiber axons derive from multiple sources in the brain stem and spinal cord neurons, including pontine nuclei, vestibular nuclei, and reticular formation, providing main excitatory input to the cerebellum by synapsing onto granule cells. The granule cells then project parallel fibers, which go up to the molecular layer, forming excitatory synapses onto Purkinje cells [126,127,128,129,130]. Parallel fibers and climbing fibers send excitatory signals to Purkinje cells, projecting their neurons to deep cerebellar nuclei neurons [131], which, in turn, give the cerebellar final output by incorporating both inhibitory and excitatory inputs from Purkinje cell axons, mossy fibers, and climbing fibers [132].

Several anatomical studies have reported that the cerebellum is interconnected with the cortex, hippocampus, and amygdala, shaping cognitive, affective, and social behaviors [14,39,133,134,135,136]. Coactivation of amygdala and cerebellum was reported during the demonstration of facial expressions in human subjects [137], and its connection with the cingulate cortex indicates participation in motivational and emotional processing [138]. Studies have reported electrical stimulation of deep cerebellar nuclei in rodents to induce the release of dopamine in the medial prefrontal cortex [139,140,141]. Previous studies have shown that the cerebellum regulates prefrontal cortical function, and disturbances in this cerebellar–prefrontal circuitry result in deficits in executive functioning and social cognition in individuals with ASD [39,142]. Overall, these reports show that the dysfunctions in the cerebellar cortical network, typically associated with ASD symptoms, could be linked to compromised connectivity between the cerebellum and cortical social areas in the brain.

Earlier studies on the cerebellum of patients with ASD reported a decrease in PC of glutamic acid decarboxylase 67 (GAD67) mRNA, a basic enzyme converting glutamate to GABA [143,144]. On the other hand, in another study, Yip et al. found increased GAD67 mRNA expression in the cerebellar molecular layer interneurons, indicating the presence of an upregulation process to counterbalance the altered inhibition of Purkinje cells [145]. Studies have reported that deep cerebellar nuclei GABAergic neurons, which project precisely to the inferior olive, showed a decrease in GAD65 mRNA expression [146,147,148]. Hence, changes in GABAergic neurotransmission in the deep cerebellar nuclei could intensely disturb Purkinje cell activity (Table 2). Other studies have also reported that serotonin concentrations were altered in the cerebellum of individuals with ASD [149,150]. Serotonin, an inhibitory neurotransmitter, plays a significant role in neurodevelopment and neuronal survival, controlling cellular migration, proliferation, neurite outgrowth, and synaptogenesis [151,152,153]. Chugani et al. showed lowered serotonin levels in the thalamus and the frontal cortex accompanying elevated serotonin concentration in the deep cerebellar nuclei, using PET scanning with tracer for serotonin synthesis in individuals with ASD [149,150]. Fatemi and colleagues reported that reelin expression was decreased in the cerebellum of individuals with ASD [143]. Reelin is a glycoprotein, encoded by the RELN gene, that can regulate the development of inhibitory synapses, proper cortex lamination, neuronal migration during development of the cerebellum and in adult life, maintaining cell signaling and synaptic function [154,155,156]. RELN genetic alteration and single-nucleotide polymorphisms (SNPs) have been reported to affect brain development and contribute to ASD [156]. Mutations in the RELN gene have been found to be associated with individuals having ASD symptoms (SFARI Gene), as postmortem brains of individuals with autism show impaired reelin signaling in the cortex and cerebellum [155]. RELN mouse mutants exhibit cerebellar hypoplasia, ataxia, reduced GC numbers, and PC migration deficits, with motor coordination and balance deficits, correlating with human RELN mutations with intense cerebellar hypoplasia [157,158]. The behavioral phenotype of RELN mutant mouse shows behavioral abnormalities that can be related to those observed in humans [159]. Mice lacking RELN C-terminal, DC-KI (C57BL/6), show increased anxiety, as well as impaired social interaction, learning, and memory, all alluding to a link between RELN and ASD behavior (Table 2) [155,159].

Multiple drugs modulating neurotransmitter functions are currently under study or at different stages of clinical trials. Baclofen and Arbaclofen, selective GABA-B agonists, appear to improve ASD-relevant behavior [160,161]. Several arbaclofen clinical studies are still ongoing with different populations and designs for ASD, reporting various outcomes, ClinicalTrials.gov registry number: NCT04271332, last update posted 30 May 2024; ClinicalTrials.gov registry number: NCT03887676, last update posted 16 July 2025. The AIMS-2-TRIALS Network (https://www.aims-2-trials.eu/ accessed on 21 May 2025) reports no significant improvement in the primary outcome measures (Socialization domain of the Vineland Adaptive Behavior Scale). However, arbaclofen showed improvement of some secondary outcomes, i.e., social interaction and communication, and repetitive behaviors/sensory profile, measured with the Social Responsiveness Scale, the Autism Impact Measure, and the Aberrant Behavior Checklist, as well as quality of life (PedsQL). The heterogeneity of the patients and protocols used in these clinical studies accounts for the variability of their results, and supports conducting larger and more standardized studies that could lead to new therapeutic approaches to improve ASD symptoms [162].

Serotonin receptor antagonists, such as Risperidone and Aripiprazole, or SSRI drugs, such as Clomipramine and Fluoxetine, are in clinical use and show improvement in aggression, anxiety, and obsessive behavior [113]. Risperidone acts both as a 5-HT2 and dopamine D2 receptor antagonist, indicating that targeting both is effective for managing repetitive, impulsive, and aggressive behavior [163,164]. Memantine, a noncompetitive NMDA antagonist, shows improvements in language function, social behavior, self-stimulatory behavior, and hyperactivity [113,161]. Several metabotropic mGluR antagonists (Fenobam, JNJ16259685, MPEP) improve social behavior and reduce repetitive behavior [113,165]. In conclusion, these findings highlight the vital role of GABAergic neurotransmission, GAD enzymes, and reelin and serotonin concentration in the cerebellum of patients with ASD; however, more studies are required to better assess the mechanisms underlying excitatory and inhibitory imbalance in ASD (Table 2).

Taken together, these observations suggest that these cerebellar processes are at the center of active chronic neural dysfunction in ASD. Elucidating potential cerebellar signaling pathways disrupted by ASD and affecting cerebellar circuitry could provide promising therapeutic interventions.

## 5. Cerebellar Signaling Involved in ASD Pathogenesis

Studies highlight the cerebellum as a pathological brain area in patients with ASD, as hundreds of identified and validated autism genes have been shown to have important functions in cerebellar development and could provide precise molecular targets for the treatment of ASD symptoms [15,166,167,168,169]. Multiple gene mutations can lead to dysregulation of signaling pathways that may affect cerebellar development, synapse function, elimination, or plasticity, and could interact to lead to variable intensity of ASD symptoms (Table 2). Manipulating these pathways may connect them more specifically to certain phenotypes and allow for mitigation of ASD symptoms.

### 5.1. Mammalian Target of Rapamycin (mTOR)

The mammalian target of the rapamycin (mTOR) signaling pathway is an important regulator with critical roles in mediating various cellular processes involving protein synthesis and synaptic plasticity [170,171]. The PI3K/Akt/mTOR pathway is expressed as two types of complexes, mTORC1, controlling cellular metabolism and autophagy, and mTORC2, controlled by TSC1/2 (tuberous sclerosis complexes 1 and 2) and upstream PI3K signaling. mTORC2 Akt phosphorylation results in mTORC1 activation. Pathogenic variants of genes of the PI-3K/Akt/mTOR signaling pathway, including FMR1, PTEN, TSC1, and TSC2, have been associated with ASD (Table 2) [114,172]. Reports on various neurodegenerative diseases show that overactivation of mTOR is associated with BBB disruption, enhanced ROS, superoxide and eNOS uncoupling, exacerbating patients’ vascular cognitive impairment and worsening the disease [173,174]. In a healthy brain, mTOR regulates BBB integrity by inhibiting the downstream effector of mTOR, rpS6, reducing superoxide production and enhancing NO production [175]. We and others have previously reported increased BBB permeability, neuroinflammation, oxidative stress, and iNOS expression in the Sema3F KO and BTBR pre-clinical models of ASD [47,176]. Multiple studies report that enhanced mTOR signaling may play a significant role in the pathophysiology of ASD and that pharmacological intervention with mTOR signaling could rescue behavioral ASD symptoms, as well as some ASD-related morphological brain changes [177,178,179]. Mitigating mTOR with rapamycin in a mouse model of Alzheimer’s disease (AD) improves cognitive function, cerebral blood flow, and microvascular endothelial function [180,181,182,183,184]. Altogether, the neuroinflammatory response associated with BBB breach, mTOR activation, and abnormal trafficking of BBB tight junction proteins could promote BBB dysfunction in ASD.

The level of cerebellar contribution to the pathogenesis of ASD still remains unclear. tuberous sclerosis complex (TSC) is a genetic disorder with increased proportions of co-morbid ASD consequences of mutation in either *TSC1* or *TSC2*, whose protein products dimerize and negatively regulate mTOR signaling [185]. A TSC2-deficient rat ASD model exhibits mTORC1 hyperactivity and enlarged cerebellar vermis white matter, thick molecular layer, and, similarly to patients with ASD or mice models, reduced PC numbers and increased numbers of astrocytes and microglia [186,187]. PC-Specific TORC1 knockout results in decreased PC number and impairs social interactions. In contrast, PC-specific TORC2 knockout results in motor coordination and gait alterations, as well as disruption of climbing fiber synapse elimination. These observations show that both of these mTOR pathway proteins are important for cerebellar development and control different ASD-related symptoms. Therefore, regulation of mTOR and its pathway is critical for cerebellar development [186]. TSC mutation is an interesting model to study the cerebellar involvement in the underlying pathogenesis of ASD, as recent reports in TSC patients reveal cerebellar pathology and associate cerebellar pathology with increased ASD symptomatology [188,189,190]. Although, *Tsc1*’s roles and its dysfunction in the cerebellum have not been well explored, Tsai et al. show that both heterozygous and homozygous loss of *Tsc1* in mouse cerebellar PCs results in autism-like behaviors, including abnormal social interaction and repetitive behavior [191]. They also reported that intraperitoneal injection of the mTOR inhibitor rapamycin in the mutant mice reversed the pathological and behavioral deficits and established critical periods of treatment to rescue specific structural and behavioral changes [192]. Thus, PC *Tsc1* mutants can provide a study model to explore the effects of PC dysfunction on neuronal networks and other mechanisms causative of ASD pathogenesis, to assess potential therapeutic strategies attenuating the effects of mTOR overactivation and BBB dysfunction. These studies could be further used to alleviate cognitive dysfunction and behavioral impairment in children with ASD. However, mTOR inhibitors also depress autophagy and have some immunosuppressive effects, impairing Treg cell differentiation; therefore, they may not be applicable to populations with ASD unless some concomitant treatment alleviates these side effects [114]. Overall, it is important to study different ASD models using the pharmacological mTOR inhibitor, rapamycin, and/or a specific amino acid diet aimed at attenuating the mTOR signaling pathway in the cerebellum.

### 5.2. Additional ASD-Linked Genes Associated with Cerebellar Development (Table 2)

Studies describing gene expression changes have been conducted in postmortem cerebellar cells or structures of patients with ASD to identify changes in known ASD-related genes, or by conducting genome-wide association studies (GWAS) to uncover region-specific novel candidate genes associated with ASD-related changes at the whole-genome level [186,193]. Among other confirmed ASD-related genes that play a role in cerebellum development and/or function, SHANK 1–3 proteins are highly expressed in the cerebellum, but differentially distributed; SHANK 1 and 2 are expressed in PC, while SHANK 2 and 3 are expressed in granule neurons [194]. Mutations or knockout of these scaffolding proteins in mice are associated with structural cerebellar changes and ASD core symptoms; knockout of Neuroligins (NLGNs) in mice cerebellum disrupts cerebellar–cortical communications and exhibits ASD symptoms; mutations of cerebellar contactin-associated protein 2 (CNTNAP2) reduce cerebellum gray matter, disrupt synapses, and show ASD symptoms; cerebellar adhesion proteins, cadherins, impair cerebellum development and are associated with ASD; the transcription factor Engrailed 2 (EN-2) is highly expressed in the cerebellum and affects connectivity and PC development, inducing ASD-related deficits. Loss of function of tuberous sclerosis complex (TSC) proteins induces cerebellar dysfunction and ASD-related social and cognitive impairment [186]. In contrast, the association of GWAS-identified genes in individuals with ASD, but not in those considered neurotypical, does not confirm that they are associated with the development of ASD symptoms. One GWAS study identified immune system genes, with particularly high cerebellar expression of sulfotransferase 1A (SULT1A) [195]; calcium-dependent activator protein for secretion-2 (CADPS2), highly expressed in the cerebellum, causes ASD [196,197]. However, some of the genes identified by GWAS, although selectively present in the cerebellum of individuals with ASD, may be associated with environmental maternal or perinatal factors for giving rise to ASD [186].

Mutations of the gene encoding the chromatin remodeler chromodomain helicase DNA-binding protein 8 (CHD8) are substantial risk factors for ASD [198,199]. Patients with CHD8 mutations regularly display cognitive deficits, gastrointestinal illnesses, anxiety, macrocephaly, and craniofacial abnormalities; in addition, CHD8 also regulates the expression of ASD risk genes associated with synaptic function and neurodevelopment [200,201,202]. A study reported by Kawamura et al. reveals that CHD8 plays a critical role in cerebellar development [203], and that the specific deletion of CHD8 in neural precursor/stem cells or granular neuron progenitor (GNP) results in cerebellar malformation and motor function defects. Additionally, they uncovered that CHD8 regulation of local chromatin accessibility modulates the expression of several neuronal genes in GNPs. Engrailed homeobox 2 (EN2) plays a crucial role in the early and late embryonic development of cerebellar neurons, as mice deficient in EN2 exhibit abnormal cerebellar development [168,204]. A few studies have found a genetic correlation between the gene encoding the transcription factor EN2 and autism, as EN2 knockout mice exhibit autism-related behavioral impairments, including social deficits and increased grooming [169,205,206,207].

Several studies have reported that transcription factors such as forkhead box 2 (Foxp2) and RAR-related orphan receptor alpha (ROR-alpha) are crucial for cerebellar PC development [208,209]. Foxp2 gene-deficient mice present with impaired cerebellar development, synaptic deficits, cell migration, and morphology, particularly affecting Purkinje cells, as well as language abnormalities, with significant alterations in vocalization [210,211,212]. Foxp2 gene mutations in humans cause developmental speech and language deficits that are associated with autism (SFARI Gene, Autism KB).

A study by Li et al. suggests that Jun proto-oncogene (JUN) and platelet-derived growth factor receptor alpha (PDGFRA) are critical genes in the cerebellum of individuals with ASD [213]. They have indicated that the activation of cerebellar JUN and PDGFRA in children with ASD may be significantly associated with inflammatory response, as they have highlighted the role of the IL17 signaling pathway in the activation of the immune response in ASD. In addition, JUN plays an important role in BBB integrity and function and in the release of a variety of inflammatory mediators, including IL-6, IL-1β, TNF-α, etc., as it regulates the transcription of proinflammatory genes [214,215].

Further investigations are needed to explore this area and provide more insights into the molecular mechanisms underlying these altered pathways in the cerebellum, and how they relate to ASD, to enable novel therapeutic strategies.

## 6. Conclusions

Several studies have reported that the cerebellum is an important brain area that affects ASD pathology [15,167,216]. Additionally, the concurrence of early behavioral defects in ASD, including motor, cognitive, and behavioral impairments, suggests that cerebellar abnormalities are important factors affecting individuals with ASD [216]. More studies need to be conducted to explore the cerebellum pathology in ASD, as well as its impaired ability to coordinate communication between neuronal groups in social, cognitive, and corticostriatal networks, thus the appearance of autism-related behaviors in children with ASD. Molecular studies to evaluate the therapeutic efficacy of different targeting interventions, including both pharmacological and dietary aspects, may offer new approaches in the management of ASD.

Further investigations are needed to better understand the complex interactions among social brain areas, connectivity, frequency bands, and physiological aspects (i.e., roles of specific cell types, maturational processes, receptors), and how they relate to different cognitive processes.

## Figures and Tables

**Table 1:** Cerebellar regions and cellular alterations in ASD may contribute to core ASD symptoms severity.

Cerebellar Area Alterations	ASD-Related Symptoms
Decreased cerebellar volume in the posterior vermis	Sensorimotor, social interaction, and communication deficits
Decreased cerebellar volume in the bilateral Crus II and right VI and Crus I/II	Social and cognitive deficits, and restricted/repetitive behavior
Reduced gray matter volume in the posterior vermis and Crus I/II	Cognition, language, social interaction, executive function and communication deficits
Reduced gray matter volume in the inferior cerebellar vermis (lobule IX), left lobule VIIIB and Crus I	Sensory over-responsiveness
Altered cerebellar activation in lobule VII and Crus I/II	Impaired connectivity with cortex, social, communication and repetitive behavior deficits
Altered white matter volume	Brain connectivity and axonal integrity linked to social, communication, and cognitive impairment
Reduction in Purkinje cells	Repetitive behavior and hyperactivity
Disruption of Granular/Purkinje cells and cerebellar deep nuclei output	Social interaction deficits, repetitive and stereotyped behavior, E/I imbalance

**Table 2:** Molecules related to autism, their localization, and autism-related effects induced by their mutation or deletion.

ASD-Related Molecules	Cerebellar Localization	Effect of Alteration in ASD
**Reelin (RELN)**	PC, internal and external granule layer neurons	PC and granular layer cell migration defects. Decreased PC numbers.
**TSC1, TSC2** **and mTOR**	Mainly in PC, but also in cerebellar interneurons	Mutations or loss of TSC in PC causes mTOR hyperactivation, impaired neural development, and social and behavioral abnormalities
**PTEN**	PC and granule layer neurons	PC-specific mutation causes PC hypertrophy, macrocephaly. Cerebellum-specific deletion impairs motor coordination, causes seizures, and granule neurons hypertrophy.
**CNTNAP2**	PC dendrites	Decreases PC dendritic morphology and complexity, and gray matter, increases excitability, impairs motor and cognitive function
**Calbindin**	PC soma and dendrites, particularly in posterior lobe, lobules VI and VI, I and Crus I/II	Decreased PC numbers, hypoplasia
**Neuroligins (NGLN)**	Posterior vermis, Crus I, mainly PC and Bergmann glia, also in stellate/basket inhibitory interneurons	Impaired cerebellar cortical communications, synaptic pruning, increased inhibitory input to PC E/I imbalance
**FMR1**	PC Crus1, Bergmann glia, granule neurons	Associated with Fragile X Syndrome. KO causes dendritic spine abnormalities, decreased primary cilia in Bergmann glia, impaired cerebellum development, and synaptic plasticity.
**SHANK 1, 2, 3**	Shank 1, 2 in PC. Shank 2, 3 in PC and granule neurons	Structural changes, impaired synaptic plasticity, reduced motor coordination, and social deficits. Disruption of excitatory synapses in PC.
**EN2**	PC, granule cells precursors	Impaired PC maturation, cerebellar foliation, development of olivocerebellar circuit, synaptic connectivity
**CHD8**	Granule neuron progenitors nuclei in external granular layer and in mature internal granular layer	Impairs activation of neurons developmental genes. Affects development, migration and proliferation of granule cells
**GABA**	PC, Bergmann and ependymal glial cells	Reduction of GAD67 in PC, of GABA receptors in glia and of transporters resulting in decreased GABA synthesis, delaying maturation of inhibitory circuits
**GAD 65/67**	Deep cerebellar nuclei, larger neurons in dentate nuclei, PC, basket cells	Decrease in deep cerebellar nuclei and PC, increase in basket cells, causing dysregulation of GABA system
**Glutamate**	PC Bergmann glia, parallel fibers	Excitotoxicity (hyper or hypoglutamergic) due mainly to loss of PC and Bergmann glia
**ROR** **α**	Stellate and basket cells and interneurons, mainly in PC	Decrease in PC, developmental and behavioral deficits
**Foxp2**	PC, and cerebellar nuclei neurons	Impaired neural maturation and connectivity, motor coordination and cognitive functions.
**MET**	Ventricular zone granule cells	Decreased number of PC, granule cells and cerebellar size. Impaired social, cognitive and motor behavior

Abbreviations: TSC: Tuberous sclerosis complex; PTEN: Phosphatase and Tensin Homolog; CNTNAP2: Contactin-associated protein 2; FMR1: Fragile X Messenger Ribonucleoprotein 1; EN2: Engrailed 2; CHD8: Chromodomain helicase DNA-binding protein 8; ROR α: Retinoic acid-related Orphan Receptor-Alpha; Foxp2: Forkhead box 2; MET: Met Proto-oncogene.

## Data Availability

No new data were created or analyzed in this study. Data sharing is not applicable to this article.
